# Screening and quantification of antibiotic residues in poultry products and feed in selected areas of Bangladesh

**DOI:** 10.14202/vetworld.2023.1747-1754

**Published:** 2023-08-28

**Authors:** Aminatu Abubakar Sani, Kazi Rafiq, Md. Tarek Hossain, Fatema Akter, Azizul Haque, Mohammad Izmal Hasan, Sabbya Sachi, Afrina Mustari, Md. Zahorul Islam, Md. Mahbub Alam

**Affiliations:** 1 Department of Pharmacology, Bangladesh Agricultural University, Mymensingh, Bangladesh; 2 Department of Pharmacology and Toxicology, Faculty of Veterinary Medicine, Usmanu Danfodiyo University, Sokoto, Nigeria; 3 Department of Physiology, Bangladesh Agricultural University, Mymensingh, Bangladesh; 4 Department of Medicine, Bangladesh Agricultural University, Mymensingh, Bangladesh

**Keywords:** antibiotic residues, broiler, high-performance liquid chromatography, layer, maximum residue limit, thin-layer chromatography

## Abstract

**Background and Aim:**

Antibiotic residues in livestock farming have been identified as a potential cause of antimicrobial resistance in humans and animals. This study aimed to determine whether antibiotic residues were present in the chicken meat, eggs, feces, and feed collected from all four districts in the Mymensingh division of Bangladesh.

**Materials and Methods:**

To detect antibiotic residues in the collected samples, qualitative thin-layer chromatography (TLC) and quantitative high-performance liquid chromatography (HPLC) were used. A total of 230 samples were analyzed for antibiotic residues of commonly used 11 antibiotics. Out of these, 40 meat and 40 feces samples were collected from broilers and layers, 30 egg samples from ducks and layers, and 120 feed samples from broilers and layers from the study area. Thin-layer chromatography was used to screen the presence of antibiotic residues; TLC-positive samples were then subjected to further HPLC analysis to determine the residue concentrations.

**Results:**

Thin-layer chromatography analysis revealed that 23.5% of the tested samples contained residues from six different antibiotic classes (tetracyclines, quinolones, beta-lactams, sulfonamides, aminoglycosides, and macrolides). Thin-layer chromatography analysis showed that 35% and 25% of the meat samples were positive for residues from the broiler and layer, respectively. About 15% and 30% of layer and duck egg samples had positive residues, respectively. Out of 120 feed samples analyzed, about 15.8% had various antibiotic residues. In addition, feces samples from broilers and layers had 50% and 35% antibiotic residues, respectively. A total of 2.5% meat and 3.3% egg samples had antibiotic residues above the maximum residue limit (MRL). Based on the findings of this study, the highest percentage of oxytetracycline, followed by doxycycline and ciprofloxacin, were detected in feed samples, and oxytetracycline was detected in meat and egg samples.

**Conclusion:**

This study clearly showed the misuse of antibiotics in the poultry sector in Bangladesh. Although antibiotic residues below the MRL level are suitable for human consumption, they may result in antimicrobial drug resistance to pathogens.

## Introduction

Antibiotics are substances or compounds, either natural, synthetic, or semi-synthetic, that prevent the growth or kill other microorganisms [1]. The development of antimicrobials and antibiotics has significantly reduced morbidity and mortality rates from many infectious diseases. It has been widely reported that indiscriminate antimicrobial use has led to antimicrobial resistance worldwide in the poultry sector and livestock, agriculture, and clinics [2–4]. Around the world, poultry farming is one of the fastest-growing industries and is the most significant protein source. However, the growing population in low-income developing countries has placed a burden on this sector due to the huge demand for cheaper dietary protein sources, which has led to the increased use of antimicrobials in poultry feed to reduce morbidity and mortality [5]. To enhance feed assimilation and reduce pathogen-related mortality, poultry producers administer antibiotics to prevent disease and promote chicken growth in the shortest possible period. The abuse of antibiotics generates drug residues in the form of metabolites or conjugates [6, 7]. This ultimately develops resistance in bacteria to these particular antibiotics, rendering the drug ineffective. To this end, the World Health Organization stated that the “misuse” of antibiotics for treatment and prophylaxis in humans, and in animals for a variety of reasons (growth promotion, prophylaxis, or therapy) is considered as the main reason for antibiotic resistance in human pathogens [1]. Antibiotics, generally broad-spectrum, reduce bacterial diversity and cause the extinction of some native species. Furthermore, by depleting the natural niches, antibiotic treatment selects resistant bacteria, increases horizontal gene transfer, and promotes pathogenic organisms. Since these changes disrupt the beneficial host-microbe relationships, it is only sensible to acknowledge the use of drugs and the consequential effect on the ecosystem [8]. Antibiotic use has been linked to increased microbial pathogen resistance and dysfunctional gut microbiota. Furthermore, a previous study by Muaz *et al*. [9] identified poultry meat as having antibiotic residues. It is thought to be one of the potential contributors to human pathogens developing antibacterial resistance. It is alarming when antibiotic residues are found in poultry meat and meat products above the maximum permitted levels. Fortunately, many researchers have observed the positive effects of temperature and refrigeration on the degradation of drug residues. Thermal treatments and sterilization have been shown to degrade residues of sulfonamides, oxytetracycline, and macrolides in edible tissues [10]. Fermentation has also been effective in lowering the residue levels of pesticides, penicillin, and several other drugs [10]. However, Vivienne *et al*. [11] noted that microwave heat may not effectively destroy high amounts of oxytetracycline, whereas normal cooking has the opposite effect. The United States Center for Veterinary Medicine has classified drug residues as it’s metabolites or compounds that persist in tissue protein sourced from animals as a consequence of the earlier usage of the drug in food animals [12]. We know that residues in meat, egg, and milk can persist even after termination of treatment for days. In meat samples, residues have been found even after 28 days, while in eggs and milk samples, they are detected up to 7 days [13]. Another study also reported the persistence of oxytetracycline, amoxicillin, and ciprofloxacin antibiotics in milk even after boiling [14]. As the presence of these residues has become a real threat, the measure of the amount of residue that is safe enough to be accepted has been a target for the production industries, especially in the developed world. This is known as the maximum residue limit (MRL). The MRLs are the maximum permissible residue levels in food products derived from animals exposed to veterinary medicine or biocidal products for animal husbandry [15]. A remarkable amount of oxytetracycline was detected in edible broiler tissues after traditional cooking [16]. However, proper maintenance of the withdrawal period minimized the amount of residues in the assessed meat samples [16]. Antibiotic use for preventive and growth-promoting purposes is already forbidden in certain affluent nations, such as Sweden, Norway, Denmark, and the European Union. In this regard, important measures for developing countries to reduce bacterial resistance to antibiotics include proper monitoring, educating farmers to keep track of withdrawal periods and restricting antibiotics use as growth enhancers [9].

Using antibiotics prudently and responsibly in animals and humans will be less likely to develop antibiotic resistance. The resistance emergence and transmission from residues necessitate research on the widespread use and misuse of antibiotics in the poultry industry. Therefore, this study assessed the use of antibiotics by farmers in the poultry sector, which can cause residues to remain in meat, eggs, feed, and feces in Mymensingh division of Bangladesh.

## Materials and Methods

### Ethical approval

The study protocol was approved by the Animal Welfare and Experimentation Ethics Committee of Bangladesh Agricultural University, Mymensingh (approval number: AWEEC/BAU/2018(31); date: December 30, 2018).

### Study period and location

The sampling period was from December 2018 to May 2019. The study was conducted in four districts of Mymensingh Division: Jamalpur, Netrokona, Mymensingh, and Sherpur in Bangladesh.

### Sample collection

A total of 230 samples from the four districts were taken from farms based on convenience sampling methods [17]. A total of 40 meat (n = 20 for broiler and layer meat), 30 eggs (n = 10 for duck and n = 20 for layer eggs), 120 different types of poultry feed samples (Broiler Starter [n = 30], Broiler Grower [n = 30], Layer Starter [n = 20], Layer Grower [n = 20], Layer Finisher [n = 20]), and 40 feces samples (n = 20 for broiler and n = 20 for layer) were collected from the study area. Meat samples were taken from freshly slaughtered birds, while egg, feed, and feces samples were taken directly from the bird’s case on the farm. Samples were collected in properly labeled air-tight zip-lock plastic bags. Collected feed samples were maintained at room temperature (25°C to 30°C), egg samples were in the refrigerator, meat, and feces were stored under −20°C in the laboratory until processing.

### Preparation of silica plates and thin-layer chromatography (TLC)

For qualitative detection of antimicrobial drug residues in samples, silica plates with 0.25 mm thickness from MN Germany were used for TLC, as described previously by Ferdous *et al*. [16] and Sattar *et al*. [18].

### Standard preparation for TLC

Antibiotic standards from HiMedia, India, were used for the detection of antibiotic residues from six different classes, namely, tetracyclines (oxytetracycline and doxycycline), fluoroquinolones (ciprofloxacin, enrofloxacin, and levofloxacin), beta-lactam (amoxicillin), aminoglycosides (gentamycin and neomycin), macrolides (erythromycin and tylosin), and sulfonamides (sulfadimidine). The purity of the antibiotic standards was 99.99%. According to our previous study in Mymensingh Division [19], 11 commonly used antimicrobial drug residues were analyzed. Standards were prepared by dissolving 0.1 g of the antibiotic into 4 mL methanol [14, 18, 20].

### Sample preparation for TLC

Feeds, droppings (feces), eggs, and meat samples were prepared and extracted using trichloroacetic acid (TCA) and diethyl ether (DEE) according to methods described elsewhere [14, 16, 18] with some modifications in the amount of phosphate buffer solution added in feed samples, that is, 10 mL instead of 5 mL. Approximately 1 g of each sample (feed, feces, muscle, and homogenized egg) was weighed, ground, blended, and transferred to a falcon tube. After homogenization, 2 mL of 30% TCA was added to the sample and mixed to enable protein precipitation, followed by 15 min of centrifugation at 4427´ *g* (Hettich D-78532, Germany). Whatman filter paper (125 mm) was used to filter the supernatant into a clean Falcon tube. An equal amount of DEE as the filtrate was added to the falcon tube. About 1 mL of the filtrate was carefully dropped onto a prepared silica plate pencil line and placed vertically into a locally prepared TLC tank with appropriate mobile phases (contained mobile phase: acetonitrile and methanol; 1:1). The silica plate in this study was dipped into a glass chamber containing a mixture of mobile phases depending on which antibiotic we were screening. The mobile phase alongside the solution mixture and the antibiotic standard were left to gradually run up the plate to approximately 90% of the height, after which it was dried and viewed under ultraviolet (UV) light (254 nm/360 nm) for any positive fluorescence compared to the standard. Polar compounds will travel short distances up the plate, while non-polar substances will diffuse into the solvent and travel further up the plate. On reaching the upper limit on the silica plate, the samples were removed and placed in the UV box for viewing [16, 18].

### Residue level quantification using high-performance liquid chromatography (HPLC)

High-performance liquid chromatography with UV detection system was used to quantify the antibiotic residue levels. C18 column at different flow rates of 1.0 mL/min for each antibiotic was used. Antibiotics were detected at 275 nm wavelength for ciprofloxacin and 260 nm wavelength for oxytetracycline and doxycycline. Each antibiotic tested was eluted using a mobile phase of 0.1% trifluoroacetic acid (TFA) in distilled water and acetonitrile. Meat samples, feeds, eggs, and feces were prepared and the deproteinized samples were extracted and put in HPLC vials, after which they were injected into the HPLC system by an auto-injector. The residue detection and quantification method was according to the protocols described elsewhere with some modifications [21, 22]. One milligram standard for each antibiotic was prepared in distilled water ranging from 50 ppm to 600 ppm (50 μg/mL–600 μg/mL) to prepare spiking solutions for the calibration curves.

### High-performance liquid chromatography working standard

Water and acetonitrile [70:30 (v/v)] were used to prepare varying concentrations of the antibiotics according to their MRL values as stock solutions and kept under −20°C. 0.2% TFA, acetonitrile, magnesium sulfate, and ethylene diamine tetra acetic acid (all from Merck, Germany), were used according to the methods described previously with modifications where applicable [22]. All reagents were of HPLC grade. Prepared stock solutions were used within 3 weeks of preparation and stored under −20°C. Maximum residue limits were considered in preparing different standards for the calibration curve of each antibiotic [22, 23]. Prepared concentration standards with respect to the MRL values were (100 mg/kg for ciprofloxacin, oxytetracycline, and doxycycline each). Based on the MRL values, concentrations of the standards were prepared from 50 μg/kg, 100 μg/kg, 200 μg/kg, 400 μg/kg, and 600 μg/kg for each antibiotic. Standards were diluted with mobile phase to have a working standard.

### High-performance liquid chromatography sample preparation

Two (2 mg/mL) of five antibiotic-free blank samples were spiked using 100 μL of different working standards, thoroughly mixed and allowed for 15 min [24]. Fortified and unknown samples were extracted and cleaned up using our modified TLC methods [16]. Working samples were individually filtered with a syringe filter (0.2 Advantec MFD, Japan).

### Fortification of samples

To study the recovery rates and linearity of samples, 2 mg/2 mL of free from any antibiotic were spiked with 100 mL of five working standards that were evenly mixed and allowed to stand at room temperature (25°C to 30°C) for 15 min. Samples were injected into the HPLC system according to previously published work with modifications where possible [22].

### Evaluation of recovery rate

As stated earlier, the following equation was used to calculate the recovery rate [21]:


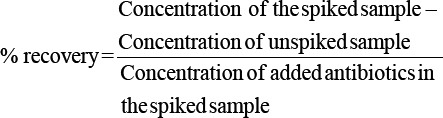


### Calibration curve

A standard solution of ciprofloxacin, oxytetracycline, and doxycycline was prepared in five different concentrations and injected into the HPLC system to obtain linear calibration curves using five points. Calibration curves were obtained using the equation *y = mx + b*; where *y* = peak area, *x* = antibiotic concentration (ppb), and the correlation coefficient (*r*²).

### Statistical analysis

The percentage of R_f_ value was analyzed using SPSS software version 26.0 (IBM Corp., NY, USA). The distance the compound moved from the baseline (where it was first spotted) divided by the distance the solvent moved from the baseline is the R_f_ factor, which was calculated and compared to the distance traveled by the standard. The data were, then, inserted into an MS Excel spreadsheet (Microsoft Excel 2018, WA, USA) for cleaning, processing, and analysis.

## Results

### Screening samples for antibiotic residues by TLC

A total of 230 samples were qualitatively analyzed by TLC for the presence of different antimicrobial residues, out of which 54 (23.5%) were confirmed to be positive. Among all TLC-positive samples, fecal samples from broiler and layer had the highest percentage of residues with 50% and 35%, respectively (Table-1). Following this, 35% of broiler meat, 30% of duck egg, and 25% of layer meat samples contained antibiotic residues. Feed provided to broiler and layer were found to have approximately 15% antibiotic residues.

**Table-1:** Percentages of TLC-positive samples for antibiotic residues.

Type of samples	Number of samples screened (n)	Positive samples n (%)	Overall positive samples n (%)
Meat			
Broiler	20	7 (35)	12 (30)
Layer	20	5 (25)	
Egg			
Duck	10	3 (30)	6 (20)
Layer	20	3 (15)	
Feed			
Broiler	60	10 (16.6)	19 (15.83)
Layer	60	9 (15)	
Feces			
Broiler	20	10 (50)	17 (42.5)
Layer	20	7 (35)	

TLC=Thin-layer chromatography

The presence of different specific antibiotic residues in various samples is shown in Table-2. Oxytetracycline was the most present antibiotic residue in all broiler, layer, and duck samples, having 12.5% in feces, 10.83% in feed, 7.5% in meat, and 6.67% in eggs. Ciprofloxacin was the next prevalent antibiotic, whose residue was also found in all samples with the highest (7.5%) in feces. Doxycycline was also found in all samples except eggs and it was the second most antibiotic (10%) detected in poultry feces. All antibiotics were spotted in poultry meat samples in different percentages except neomycin. In eggs, 6.67% oxytetracycline and ciprofloxacin were detected. Neomycin was found only in layer eggs (3.33%), tylosin in broiler meat (2.5%), and levofloxacin in layer meat (2.5%).

**Table-2:** Percentages of different antibiotics residue detected by TLC.

Type of samples (n)	Antibiotics								

	O	CIP	AMX	EX	SDD	DO	TL	LE	N
Meat (40)									
Broiler (20)	2	2	1	-	-	1	1	-	-
Layer (20)	1	-	1	1	1	-	-	1	-
Percentage	7.5	5	5	2.5	2.5	2.5	2.5	2.5	
Egg (30)									
Duck (10)	1	-	1	1	-	-	-	-	-
Layer (20)	1	-	1	-	-	-	-	-	1
Percentage	6.67		6.67	3.33					3.33
Feed (120)									
Broiler (60)	7	1	-	-	-	1	-	-	-
Layer (60)	6	2	-	-	-	2	-	-	-
Percentage	10.83	2.5				2.5			
Feces (40)									
Broiler (20)	3	3	1	1	-	2	-	-	-
Layer (20)	2	-	2	-	1	2	-	-	-
Percentage	12.5	7.5	7.5	2.5	2.5	10			

O=Oxytetracycline, CIP=Ciprofloxacin, AMX=Amoxicillin, EX=Enrofloxacin, SDD=Sulfadimidine, DO=Doxycycline, TL=Tylosin, LE=levofloxacin, N=Neomycin

### Quantification of antibiotic residues by HPLC

Samples that were positive for antibiotic residues in TLC were further analyzed by HPLC for their concentration. Table-3 shows the concentration of antibiotic residues in meat and egg samples. In broiler meat, oxytetracycline residue concentration was 278.96 μg/kg which exceeded the MRL value (200 μg/kg). Oxytetracycline residue concentration in duck eggs was 443 μg/kg and in layer eggs was 211.53 μg/kg, whereas in duck eggs, it was higher than the MRL (400 μg/kg). The concentration of neomycin was 267.77 μg/kg in layer eggs. In meat, 2.5% of samples exceeded the MRL which was 3.3% in case of eggs.

**Table-3:** Concentration of antibiotics residue of TLC-positive meat and egg samples analyzed by HPLC with MRL values.

Type OF Samples (n)	Conc. of antibiotics residue (µg/kg) of TLC-positive samples analyzed by HPLC with MRL values									No. of samples above MRL	Overall percentage

	O Conc. (MRL)	CIP Conc. (MRL)	AMX Conc. (MRL)	EX Conc. (MRL)	SDD Conc. (MRL)	DO Conc. (MRL)	TL Conc. (MRL)	LE Conc. (MRL)	NEO Conc. (MRL)		
Meat (40)											
Broiler (20)	278.96 35.37 (200)	0.29 BDL (100)	3.89 (50)	-	-	12.73 (100)	BDL	-	-	1	2.5
Layer (20)	73.47 (200)	-	BDL	BDL	BDL	-	-	5.01 (100)	-		
Egg (30)											
Duck (10)	443.00 (400)	-	BDL	75.00 (100)	-	-	-	-	-	1	3.3
Layer (20)	211.53 (400)	-	1.02	-	-	-	-	-	267.77 (500)		

n=number of samples analyzed by HPLC, TLC=Thin-layer chromatography, HPLC=High-performance liquid chromatography, MRL=Maximum residue limit, O=Oxytetracycline, CIP=Ciprofloxacin, AMX=Amoxicillin, EX=Enrofloxacin, SDD=Sulfadimidine, DO=Doxycycline, TL=Tylosine, LE=Levofloxacin, N=Neomycin, BDL=Below detectable limit

The concentration of antibiotic residues in poultry feed and feces samples is shown in Table-4. In feed samples, oxytetracycline residue concentration was 200.35 μg/kg in the layer starter and 214.52 μg/kg in layer grower, while doxycycline residue concentration was 126.91 μg/kg in the layer starter samples. In broiler starters and growers, oxytetracycline was found as 104.86 and 112.69 μg/kg, respectively. Ciprofloxacin residue concentration was 184.74 μg/kg in the layer finisher. From TLC-positive feed samples, 11.67% of broiler feeds and 15% of layer feed samples were identified as residues positive by HPLC. Five out of six TLC-positive antibiotics were detected in poultry feces samples except sulfadimidine. Oxytetracycline residue concentration was 150.21 μg/kg in broiler feces. About 45% of broiler and 30% of layer feces samples were HPLC-positive.

**Table-4:** Concentration of antibiotics residue of TLC-positive feed and feces samples analyzed by HPLC.

Type of samples	Conc. of antibiotics residue (µg/kg) of TLC-positive samples analyzed by HPLC						No. of HPLC Positive Samples	Percentage	Overall Percentage

	O Conc.	CIP Conc.	AMX Conc.	EX Conc.	SDD Conc.	DO Conc.			
Feed (n = 120)									
Broiler Starter (n = 30)	BDL 4.90 104.86	-	-	-	-	0.50	3	11.67	13.33
Broiler Grower (n = 30)	30.49 2.53 112.69 BDL	5.80	-	-	-	-	4		
Layer Starter (n = 20)	200.35 94.39	-	-	-	-	9.85	3	15	
Layer Grower (n = 20)	214.52 6.65 11.73	BDL	-	-	-	126.91	4		
Layer Finisher (n = 20)	1.56	184.74	-	-	-	-	2		
Feces (n = 40)									
Broiler (n = 20)	150.21 BDL 97.14	123.59 10.51 28.69	75.11	122.39		23.17 0.02	9	45	37.5
Layer (n = 20)	37.43 77.08		0.25 6.98		BDL	3.59 21.38	6	30	

n=Number of samples analyzed by HPLC, TLC=Thin-layer chromatography, HPLC=High-performance liquid chromatography, O=Oxytetracycline, CIP=Ciprofloxacin, AMX=Amoxicillin, EX=Enrofloxacin, SDD=Sulfadimidine, DO=Doxycycline, BDL=Below detectable limit

## Discussion

The study aimed to identify and quantify the presence of specific antibiotic residues in poultry industry of Mymensingh Division. Thin-layer chromatography was used to detect the presence of residues from various samples, which were further quantified using HPLC. This study is crucial because antibiotic residues or metabolites can remain in animal proteins such as meat, egg, and milk after a prior drug use [12] and even after termination of the recommended dosage [13]. In this study, of the 230 samples tested, 54 (23.4%) were positive for residues, about a quarter of all tested samples. This is an important finding and a matter of concern as the presence of a trace of residues can cause havoc to public health due to the high risk of developing antimicrobial resistance.

Poultry meat is a good substitute for red meat due to its nutritional value, price, and availability. However, a decrease in poultry meat quality has recently been observed due to uncontrolled drug use, inaccurate biosafety measures, and lack of maintenance of the withdrawal period [25]. When studying broiler meat, we found only 1 (2.5%) sample to harbor residues above the MRL (200 mg/kg) of oxytetracycline from the 12 residue-positive meat samples (Table-3). However, the other samples with residues were below MRL. Oxytetracycline levels in chicken muscles were also found to be below the MRL values [26]. About 20% of ciprofloxacin, enrofloxacin, and amoxicillin contamination of broiler meat were also traced in Mymensingh Division, Bangladesh [27]. In this regard, chicken meat from different regions of Lebanon contained the highest 32.5% of ciprofloxacin (quinolone) residues, followed by tetracyclines (17.5%) [22]. Tetracycline residues were the most common antibiotic residues among poultry farmers [28]. In the current study, we also observed that oxytetracycline was the most prevalent (7.5%) residue in poultry meat, followed by ciprofloxacin (5%) and amoxicillin (5%) (Table-2). In this study, farmers used more of the tetracycline group of antibiotics followed by ciprofloxacin, probably because they are easily accessible and cheaper. This could be a pointer to the fact that many susceptible organisms no longer respond to tetracycline treatments, even in humans [29]. Moreover, carcinogenic effects have also been reported from the residues of antibiotics such as oxytetracycline, sulfamethazine, and furazolidone [29].

Since practically everyone consumes poultry eggs worldwide, the presence of antibiotic residues in eggs is a serious public health concern. In this study, 6 (20%) of the total eggs were residue positive from duck and layer birds (Table-1). Oxytetracycline and amoxicillin were detected in layer and duck eggs (6.67%), whereas enrofloxacin and neomycin were found only in one species (3.33%). We did not find ciprofloxacin in eggs, which is contrary to the findings of Billah *et al*. [30], who detected a high prevalence of ciprofloxacin in layers eggs. When quantified by HPLC, we found that 1 (3.3%) duck egg sample had oxytetracycline residues above the MRL value (400 μg/kg) (Table-3). In another study, 47.3% of the 146 meat and egg samples contained residues, whereas 11.6% of the samples had residues that exceeded the MRL value [31]. Other studies have also observed the presence of antibiotic residues in poultry eggs [32–36], some of which also noted the distribution of residues in egg white and yolk. Interestingly, trying different storage conditions (room temperature or refrigeration at 4°C) and 10 min of boiling could not minimize the amount of antibiotic residues in eggs [36]. This scenario explains the concern, which otherwise could result in serious public health hazards.

Indiscriminate use of antibiotics in poultry feed is another big problem that we face nowadays. In Bangladesh, antibiotics are regularly used as feed additives at sub-therapeutic dose for growth promotion and prophylaxis [27, 37]. However, these antibiotic residues could find their way into consumer food products and the human food chain. Poor record keeping can make the problem worse, if farmers sell the birds and their products without completing specified withdrawal periods. In this study, among 120 poultry feed samples, 19 (15.83%) were residue positive with 13 (10.83%) oxytetracycline, 3 (2.5%) doxycycline, and 3 (2.5%) ciprofloxacin (Table-2). Another study uncovered that about 18.89% of poultry feed samples were enriched with antibiotic residues in Bangladesh [38]. Other researchers have also found the presence of antibiotic residues in poultry feed, including enrofloxacin (46.67%), ciprofloxacin (30.00%), and amoxicillin (23.33%) [27]. Farmers prefer antibiotic growth promoters in poultry feed to ensure a profitable production system [39]. However, this poses major health risks due to the development of antibiotic-resistance genes, making it difficult to treat humans and animals [40]. Another major issue is the possibility of the horizontal spread of antibiotic resistance genes from human to animal pathogens and vice versa [41]. Therefore, it is essential to raise public knowledge about the misuse of antibiotics in chicken feed as a growth enhancer.

Another threat of using random antibiotics in poultry sector is their excretion through feces. Unmetabolized residues passing through feces could generate antimicrobial resistance to the organisms living in soil and surfaces. In this study, 17 (42.5%) out of 40 fecal samples from broiler and layer were positive for residues, where oxytetracycline was the highest (12.5%) (Table-1). In another study, chlortetracycline residues in poultry feces were identified up to 25 days post-treatment [42]. Furthermore, in line with our findings, Van Epps and Blaney reported about 25% of tested fecal samples were positive for antibiotic residues of enrofloxacin (quinolone) from China, Egypt, and Austria [43]. Fertilization of soil with poultry manure having antibiotic residues also results in the contamination of vegetation with antibiotics. Unpublished data in our research group detected oxytetracycline and ciprofloxacin residues in different vegetables collected from field fertilized with poultry feces [44]. Therefore, it is crucial to maintain proper guidelines in using antibiotics in the poultry sector, following withdrawal periods, and maintaining and disposing of poultry manure.

### Limitations

The study was limited to some selected parts and not all of the Mymensingh Division. The sample size was also small because we were led by volunteers and government representatives rather than being familiar with all farms in the study area.

## Conclusion

The findings of this study, point out a high threat posed by the use and misuse of antimicrobials in poultry production, even though most of them were below the MRL values. There is an obvious trend of use and misuse of antibiotics in the poultry sector, contributing to the menace of antimicrobial resistance. Policymakers should take proper measures and ensure compliance through surveillance and testing of poultry products regularly throughout the country.

## Authors’ Contributions

KR, MTH, and AAS: Conceptualized and supervised the study. KR, AAS, MTH, SS, and AM: Performed all the experiments, and prepared the manuscript draft. KR, FA, and MMA: Designed and supervised the study and revised and finalized the manuscript draft. AAS, AH, MIH, FA, and MZI: Performed the statistical analysis and prepared the graphs and tables. KR, AAS, and FA: Revised the manuscript. All authors have read, reviewed, and approved the final manuscript.
